# Reducing employee turnover in hospitals: estimating the effects of hypothetical improvements in the psychosocial work environment

**DOI:** 10.5271/sjweh.3969

**Published:** 2021-08-31

**Authors:** Jimmi Mathisen, Tri-Long Nguyen, Johan Høy Jensen, Reiner Rugulies, Naja Hulvej Rod

**Affiliations:** Department of Public Health, University of Copenhagen, Copenhagen, Denmark; Department of Occupational and Environmental Medicine, Copenhagen University Hospital, Bispebjerg Hospital, Copenhagen, Denmark; Copenhagen Stress Research Center, Copenhagen, Denmark; National Research Centre for the Working Environment, Copenhagen, Denmark; Department of Psychology, University of Copenhagen, Copenhagen, Denmark

**Keywords:** Key terms employee exit, health care, health services research, hospital staff, occupational health, parametric g-formula, simulation study

## Abstract

**Objectives:**

Poor psychosocial work environments in hospitals are associated with higher employee turnover. In this prospective cohort study, we aimed to identify and quantify which aspects of the psychosocial work environment have the greatest impact on one-year employee turnover rates within a hospital setting, both overall and within occupational groups.

**Methods:**

The study population included 24 385 public hospital employees enrolled in the Danish *Well-being in Hospital Employees* cohort in 2014. We followed the participants for one year and registered if they permanently left their workplace. Using baseline sociodemographic, workplace, and psychosocial work environment characteristics, we applied the parametric g-formula to simulate hypothetical improvements in the psychosocial work environment and estimated turnover rate differences (RD) per 10 000 employees per year and 95% confidence intervals (95% CI).

**Results:**

Of the 24 385 participants, 2552 (10.5%) left the workplace during the one-year follow-up. Up to 44% of this turnover was potentially preventable through hypothetical improvements in the psychosocial work environment. The specific hypothetical improvements with the largest effects were in satisfaction with work prospects (RD -522 turnovers per 10 000 person-years, 95% CI -536– -508), general job satisfaction (RD -339, 95% CI -353– -325) and bullying (RD -200, 95% CI -214– -186). The potential for preventing turnover was larger for nurses than for physicians and other healthcare employees.

**Conclusions:**

Improvements in the psychosocial work environment may have great potential for reducing turnover among hospital staff, particularly among nurses.

High employee turnover in hospitals can hamper workflows (1), lead to lower quality of care (2–4) and incur considerable financial expenses (1, 5, 6). However, retaining qualified employees in healthcare is a challenge faced by many countries (7). In Denmark, the US, Canada and Australia, yearly hospital employee turnover rates as high as 15–27% have been reported (8–11).

Stressful working conditions in hospitals may lead to higher turnover rates. Previous studies have shown that psychosocial work environment factors including lack of social support, low leadership quality, bullying and violence, as well as potential cognitive and emotional reactions to these factors such as stress and low job satisfaction, are associated with higher employee turnover among hospital staff (1, 4, 9, 12–15). Therefore, interventions aimed at improving the psychosocial work environment in hospitals hold the potential for reducing employee turnover rates.

Such interventions should be guided by evidence about the relative importance of different psychosocial work environment aspects for employee turnover. Typically, quantitative research studies measure the effects of psychosocial exposures by, for example, reporting regression coefficients. These are useful estimates of the average risks of outcomes in individuals. However, such coefficients provide little information about potential changes in the outcome if population exposure levels were changed and are therefore arguably less useful to estimate the effects of potential interventions and improvements at the population level (16, 17).

In recent years, the parametric g-formula has been increasingly used to estimate population-level effects of changes in exposures (16–19). The parametric g-formula is a method based on regression models. However, instead of reporting regression coefficients, it is used to estimate potential changes in the outcome by setting a contrast between two scenarios for the same population via simulations: for example, this population being exposed versus this population being unexposed (20–22). The parametric g-formula thereby enables the design of hypothetical population-level experiments using observational data. Such an experiment could, for example, include estimating the difference in outcomes (such as turnover rates) between a scenario where all employees were to receive a hypothetical improvement in exposure (such as high supervisor support) and a scenario where no employee was to receive the exposure (such as no supervisor support). As such, the parametric g-formula is better suited to estimate the population-level impact of interventions and improvements than regression coefficients.

In this prospective cohort study, we apply the parametric g-formula to simulate hypothetical improvements in a wide variety of work environment factors in a large and comprehensive cohort of hospital employees. We aim to identify and quantify which aspects of the psychosocial work environment hold the greatest potential for reducing the one-year employee turnover rate, overall and within different hospital occupational groups.

## Methods

### Study population

**Figure 1:**
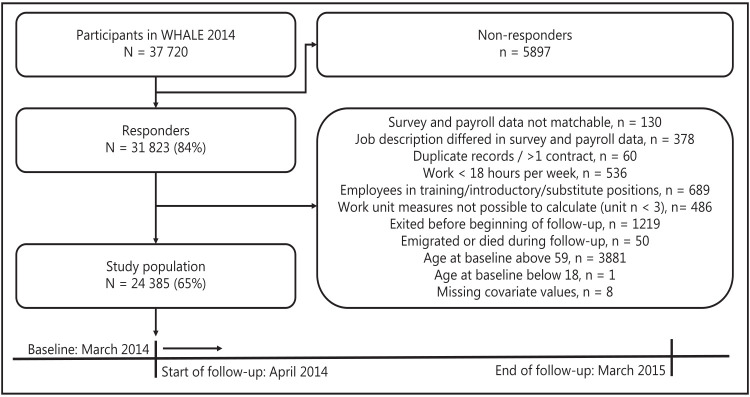
Study population, exclusion criteria, and follow-up period.

The study population included participants in the 2014 wave of the *Well-being in Hospital Employees* (WHALE) cohort (23). The cohort contains self-reported workplace survey assessments and monthly updated administrative data from all employees in the Capital Region of Denmark (public healthcare enterprise) employed in March 2014. Of the 37 720 employees invited to the survey, 31 823 (84%) responded. We excluded responders based on several criteria (figure 1). Among other criteria, responders were excluded if they had left the region before the start of follow-up (N=1219) or were employed in temporary positions (N=689). Senior employees (age >59 years, N=3881) were also excluded to omit turnover due to statutory or early retirement. The final study population included 24 385 employees.

### Psychosocial work environment

**Figure 2:**
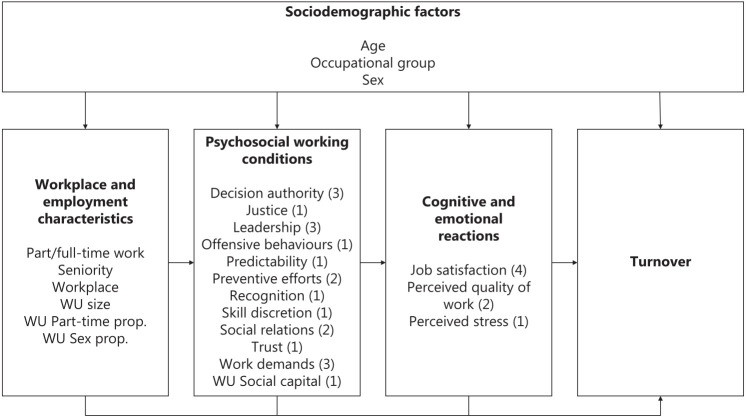
Conceptual model depicting the assumed associations between covariate domains. The number of items within each covariate category is shown in parenthesis. [WU=work unit.]

The WHALE cohort contains rich information on numerous psychosocial work environment factors that are associated in complex ways. To inform the analyses, we organized the different aspects of the psychosocial work environment by adapting a framework for research into psychosocial work environments and health. The original framework includes seven hierarchically ordered domains, from macro-level socioeconomic structures to individual health outcomes (24). Here, we employed roughly the same structure (figure 2). Our conceptual model includes three hierarchically ordered domains: (i) workplace and employment characteristics, (ii) psychosocial working conditions, and (iii) cognitive and emotional reactions. Each domain includes several constructs, and each construct may include several items. We treated each item separately because the individual items within the constructs may conceptually differ and may influence the probability of turnover differently. The model describes a unidirectional process in which each domain can influence the probability of turnover directly or indirectly through other domains lower in the hierarchy. We assume that demographic factors (age, sex, and occupational group) can influence all other domains.

Information on sociodemographic factors, and workplace and employment characteristics were derived from administrative data at baseline. Information on psychosocial working conditions and cognitive and emotional reactions were derived from self-reported responses to 39 items (mostly on Likert scales). Of these, 23 items were derived from the Copenhagen Psychosocial Questionnaire II (COPSOQ II) (25). We included 20 of the 32 psychosocial working conditions and all seven cognitive and emotional reactions in the statistical models. The selection procedure is described below. Measurement and operationalization details are presented in the supplementary material (www.sjweh.fi/article/3969), appendix 1.

### Turnover

We defined the rate of turnover as the proportion of employees who permanently left the Capital Region of Denmark within a one-year follow-up period spanning from April 2014 to March 2015 (figure 1). Employees who permanently left were identified in the administrative data by registration of no wage-related affiliation in a specific month accompanied by a status code indicating permanent exit.

### Analytical framework

We used the parametric g-formula to estimate changes in turnover rates resulting from simulated improvements in psychosocial work environment factors. Briefly, we first constructed regression models to predict turnover from the included covariates. Then, we simulated hypothetical improvements in psychosocial work environment factors in the data and used the regression model coefficients to predict turnover rates conditional on these improvements. The procedure relies on the assumption of exchangeability (no unmeasured confounding) like other methods for analyzing observational data. Software codes for applying the parametric g-formula can be obtained via Hernán & Robins (22).

To reduce noise and overfitting of the regression models, we initially excluded psychosocial working condition covariates that had limited association with turnover (26). We assessed the deviance in turnover explained by each covariate using Type 2 ANOVA and excluded 12 covariates that explained less than 0.25% of total model deviance. Among these covariates were social support from colleagues and supervisors and having been exposed to threats, acts of violence, or sexual harassment within the last 12 months (supplementary table S1). As these covariates were only weakly associated with the overall turnover rate, they were unlikely to be important exposures or confounders.

Guided by the conceptual framework, we constructed two regression models to predict the individual one-year probability of turnover: The first model predicted turnover based on psychosocial working conditions adjusted for sociodemographic factors, and workplace and employment characteristics. The second model predicted turnover based on cognitive and emotional reactions adjusted for sociodemographic factors, workplace and employment characteristics and psychosocial working conditions.

We fitted the regression models using the group-lasso interaction network algorithm (‘glinternet’ package in R) (27). This algorithm uses the LASSO procedure to reduce overfitting while automatically identifying and modelling all relevant first-order interactions. Thereby, we modelled the potentially heterogeneous associations between the covariates and turnover across occupational groups. Continuous variables were handled using fractional polynomials to accommodate non-linearity (28).

To assess the generalizability of the models, we applied internal-external cross-validation (29). This procedure is an extended and strong form of internal validation that can exploit the organizational structure of the Capital Region of Denmark to evaluate generalizability. Briefly, following a leave-one-out principle, a prediction model was derived using data from 12 out of 13 organizations and then validated in the omitted (“external”) organization. This procedure was repeated 13 times, omitting each organization once. Summary measures were then used to assess the generalizability of the prediction models across the organizations. The potential heterogeneity was accounted for by including a workplace covariate in the regression models (30).

Finally, we applied the parametric g-formula to estimate changes in turnover rates from simulated improvements in psychosocial working conditions and cognitive and emotional reactions. First, we averaged the predicted individual probabilities of turnover into an estimated turnover rate. Then, we estimated changes in this turnover rate under different simulated scenarios. We constructed the scenarios by first creating two copies of the original dataset and then simulating that employees took different covariate values within each copy. Next, we estimated the average turnover rate in each copy using coefficients from the regression models. The contrast between these rates was calculated and expressed as the rate difference of turnover per 10 000 employees per year (RD). We estimated 95% confidence intervals (95% CI) by bootstrapping each estimate 1000 times.

We estimated two types of contrasts: dichotomous contrasts, which compared scenarios where all employees were set to have the most desirable covariate level, versus scenarios where all employees were set to have the least desirable covariate level. The most desirable covariate levels were defined as ‘high’ for all covariates measured on Likert scales (high; medium; low), ‘none’ for a measure of perceived stress (none; low; high), ‘no’ for dichotomous measures of offensive behaviors and ‘yes’ for a dichotomous measure of having had a performance and development review within the last 12 months. The least desirable level was defined as the reversed levels (low; high; yes; no) (see supplementary appendix 1 for details). These dichotomous contrasts emulated the effects from a hypothetical randomized controlled trial (‘treatment’ versus ‘no treatment’). Also, we estimated cohort-specific contrasts which compared scenarios where all employees were set to have the most desirable covariate level (as described above) versus where all employees were set to have their actually observed covariate level. These contrasts emulate the effects of a hypothetical intervention that would remove a given exposure from a real-world setting, similar to population attributable fractions (17). For both contrast types, we simulated improvements in each covariate individually, as well as in all covariates simultaneously. The contrasts were estimated for all employees combined and each occupational group separately. All analyses were conducted in R version 4.0.2 (31).

## Results

Of the 24 385 employees, 2552 (10.5%) left the Capital Region of Denmark during the one-year follow-up period. The characteristics of the study population in terms of sociodemographic factors and workplace and employment characteristics are shown in table 1. The turnover rate was highest among physicians and lowest among service employees. Furthermore, the turnover rate was lower among older employees and those with higher seniority.

**Table 1:** Characteristics of the study population in terms of socio- demographic factors and workplace and employment characteristics.

	N	%	Exit (N)	Exit (%)
Total	24 385	100	2552	10.5
Sociodemographic factors				
Age, years ^a^				
18–34	5485	22.5	834	15.2
35–44	7481	30.7	941	12.2
45–54	8167	33.5	638	7.8
55–58	3252	13.3	166	5.1
Sex				
Female	19 408	79.6	2054	10.6
Male	4977	20.4	498	10.0
Occupational group				
Physicians	2154	8.8	315	14.6
Nurses	8768	36.0	943	10.8
Other healthcare employees ^b^	5507	22.6	507	9.2
Pedagogical employees	587	2.4	73	12.4
Service employees	2647	10.9	215	8.1
Administrative leaders	292	1.2	26	8.9
Administrative employees	4430	18.2	473	10.7
Workplace and employment characteristics				
Seniority, years ^a^				
<2	4466	18.3	864	19.3
2–4	4457	18.3	579	13.0
5–9	7197	29.5	708	9.8
10–14	3372	13.8	223	6.6
15–24	2940	12.0	122	4.2
≥25	1953	8.0	56	2.2
Part/full-time work ^c^				
Part-time	8287	34.0	892	10.8
Full-time	16098	66.0	1660	10.3
Number of employees in work-unit ^a^				
0–13	8116	33.3	852	10.5
14–23	7972	32.7	780	9.8
≥24	8297	34.0	920	11.1
Proportion of females in work unit (%) ^a^				
0–75	7503	30.8	822	11.0
76–95	8623	35.4	915	10.6
96–100	8259	33.9	815	9.9
Proportion of part-time workers in unit (%) ^a^				
0–15	7726	31.7	821	10.6
15–50	9090	37.3	944	10.4
50–100	7569	31.0	787	10.4

a These covariates are categorized only for descriptive purposes; they are handled as continuous covariates in the regression models.

b ‘Other healthcare employees’ include nursing assistants, physiotherapists, midwives, biomedical laboratory employees, occupational therapists and radiographers.

c Full-time employment is defined as 37 hours per week or more. Part-time employment is defined as less than 37 hours per week.

Distributions of the psychosocial working conditions and the cognitive and emotional reactions are shown in supplementary appendix 2. The regression models displayed good predictive performance (C-statistics: Model 1: 0.74; Model 2: 0.76) as well as reasonably good performance and generalizability across organizations (see supplementary appendices 3 and 4).

**Figure 3:**
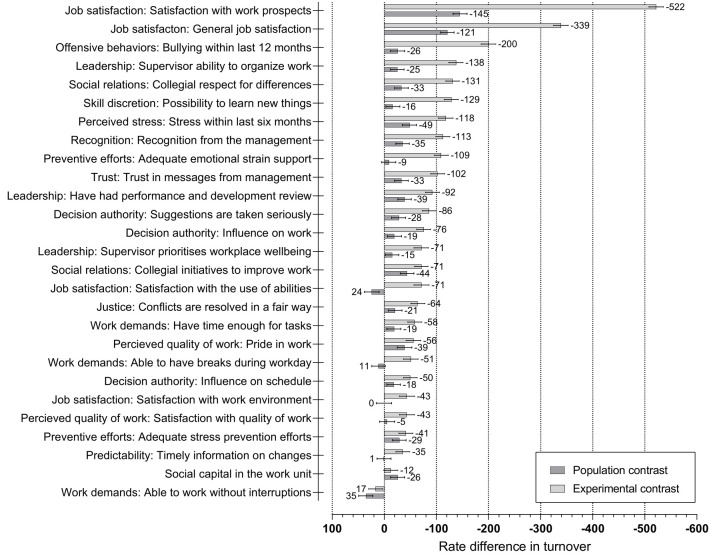
Dichotomous and cohort-specific rate differences in turnover per 10 000 employees per year and 95% confidence intervals associated with hypothetical improvements in psychosocial working conditions^a^ and cognitive and emotional reactions^b^ among all employees. Rate differences and confidence intervals are reported in supplementary table S3. ^a^ Adjusted for sociodemographic structures, and workplace and employment characteristics. ^b^ Adjusted for sociodemographic structures, workplace and employment characteristics, and psychosocial working conditions.

There were an estimated 1047 (95% CI 1037–1056) employees leaving per 10 000 employees per year (similar to the observed turnover rate). Estimated rate differences resulting from hypothetical improvements in psychosocial working conditions and reactions are shown in figure 3. All rate differences and 95% CI for all employees combined and for individual occupational groups are presented in supplementary appendix 5.

The largest dichotomous rate differences in turnover (the most desirable versus least desirable scenarios) were estimated for hypothetical improvements in satisfaction with work prospects (RD -522 turnovers per 10 000 person-years, 95% CI -536– -508) and general job satisfaction (RD -339, 95% CI -353– -325). Among the psychosocial working conditions, hypothetical improvements in bullying (RD -200, 95% CI -214– -186), supervisor ability to organize work (RD -138, 95% CI -151– -124), collegial respect for differences (RD -131, 95% CI -144– -118), and the possibility to learn new things (RD -129, 95% CI -142– -115) had the strongest associations with turnover rates.

The cohort-specific rate difference (the most desirable versus observed scenarios) of all improvements simultaneously was 456 fewer turnovers per 10 000 person-years (95% CI -467– -445) corresponding to a 44% reduction in the turnover rate. This reduction represents an upper bound of the proportion of turnover that could potentially be prevented through improvements in the psychosocial working environment in this specific population. The cohort-specific rate differences of individual improvements were substantially smaller than the corresponding dichotomous rate differences, indicating that the psychosocial work environment was already relatively well-functioning in this population of Danish hospital employees. Individual hypothetical improvements in satisfaction with work prospects (RD -145, 95% CI -159– -132) and general job satisfaction (RD -121, 95% CI -134– -108) had the strongest associations with turnover. Among the psychosocial working conditions, hypothetical improvements in collegial initiatives to improve work (RD -44, 95% CI -57– -31), having had a performance and development review within last 12 months (RD -39, 95% CI -52– -26), and recognition from the management (-35, 95% CI -48– -22) showed the strongest associations with turnover. A few hypothetical improvements were associated with a slightly higher turnover rate, for example, being able to work without interruptions (RD 35, 95% CI 22–49).

**Figure 4:**
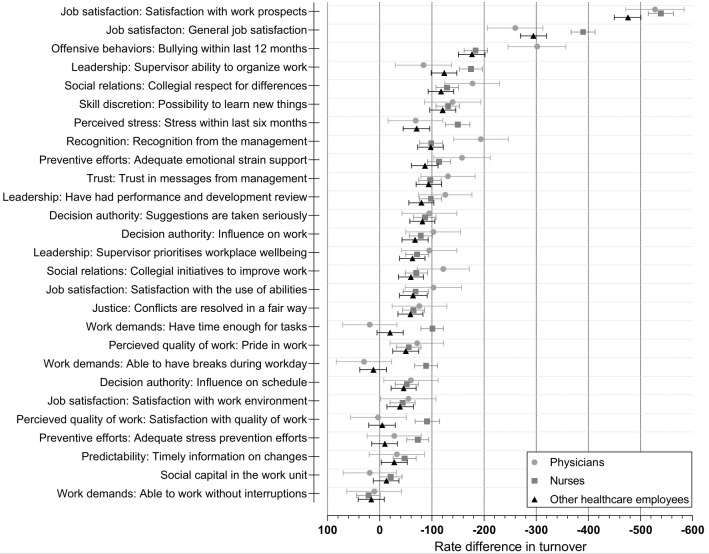
Dichotomous rate differences in turnover per 10 000 employees per year and 95% confidence intervals associated with hypothetical improvements in psychosocial working conditions^a^ and cognitive and emotional reactions^b^ among physicians, nurses and other healthcare employees. Rate differences and confidence intervals are reported in supplementary tables S4-S6. ^a^ Adjusted for sociodemographic structures and workplace characteristics. ^b^ Adjusted for sociodemographic structures, workplace characteristics, and psychosocial working conditions.

Dichotomous rate differences among physicians, nurses, and other healthcare personnel, respectively, are shown in figure 4. Hypothetical improvements in all psychosocial working conditions and reactions simultaneously were associated with a larger turnover reduction among nurses (RD -2357, 95% CI -2383– -2331) than physicians (RD -2118, 95% CI -2177– -2060) and other healthcare employees (RD -1742, 95% CI -1772– -1711) (supplementary tables S4–S6). Some individual hypothetical improvements were also more strongly associated with lower turnover among nurses than physicians or other healthcare employees. These included general job satisfaction, supervisor ability to organize work, satisfaction with quality of work as well as several improvements related to stressful work environments (stress within last six months, having enough time for tasks, able to have breaks during workday). Conversely, hypothetical improvements in bullying and recognition from the management were more strongly associated with turnover among physicians. Hypothetical improvements among other healthcare employees showed associations with turnover similar to doctors and/or nurses in most cases. Associations for the remaining occupational groups are shown in supplementary tables S7 – S10.

## Discussion

We found that 2552 hospital employees (10.5%) left the Capital Region of Denmark within the one-year follow-up and that 44% of this turnover could potentially be prevented through improvements in the psychosocial work environment. Hypothetical improvements targeting job satisfaction elements, especially satisfaction with work prospects and general job satisfaction had the largest estimated impact on turnover, while hypothetical improvements in bullying, collegial respect for differences, supervisor ability to organize work and the possibility to learn new things were estimated to have a moderate impact. Hypothetical improvements in psychosocial work environment factors were estimated to have a larger impact on turnover among nurses than among physicians and other healthcare employees.

### Comparison with previous work

To our knowledge, this is the first study to investigate changes in turnover rates associated with hypothetical improvements in the psychosocial working environment among hospital staff, making it difficult to compare directly with previous literature. However, the findings can be indirectly compared to studies of associations between psychosocial work environment factors and turnover, and with findings from actual interventions. In line with our findings, elements of job satisfaction have been identified as strong determinants of turnover (1, 4, 15). The association between turnover and other psychosocial work environment factors, such as leadership quality, recognition, trust, bullying and work stress has also been found in previous studies (1, 4, 13, 15). In addition, interventions targeting the improvement of leadership practices and teamwork can decrease turnover among nurses (32). We add to this literature by quantifying and comparing the potential importance of each of these factors for turnover in a large data sample with comprehensive coverage of various domains of the working environment.

Some previously reported determinants of turnover, such as social support from colleagues and supervisors, and exposure to threats, violence, or sexual harassment (1, 4, 13, 15), were only weakly associated with turnover in our study. Nonetheless, they are likely highly important for the psychosocial work environment of individual employees.

Previous studies have often investigated associations between the psychosocial work environment and intentions to leave and not actual turnover behavior (1, 4, 13). While intentions to leave is a good predictor of eventual turnover, the two only moderately correlate (15, 33). Associations between psychosocial work environment factors and turnover intentions are generally stronger than for actual turnover (15).

Most previous studies have been conducted among either nurses or doctors, but not both groups at the same time. Some studies have included hospital employees more generally but have not investigated whether associations were heterogeneous across staff types (9, 14). Therefore, to the best of our knowledge, the findings of heterogeneous associations between psychosocial work environment factors and turnover in different occupational groups are novel. Our findings suggest that in regard to turnover, nurses are more responsive to adverse psychosocial working environments compared with physicians and other healthcare employees, especially regarding factors related to work stress. Conversely, bullying and recognition appeared to be more important for turnover among physicians than among the other groups. These findings suggest that psychosocial work environment interventions aiming to reduce turnover should consider that interventions on specific factors might have different effects in different occupational groups.

### Interpretation

We found that job satisfaction is one of the most important aspects of the psychosocial working environment in relation to turnover. Job satisfaction reflects a general attitude towards one’s job, and this attitude is in itself associated with many work environment factors (34, 35). Because job satisfaction and psychosocial working conditions are tightly intertwined, and because all covariates were measured at baseline, it is difficult to completely separate their effects. Also, satisfaction with one’s job is a psychological state that might be difficult to intervene on directly. Interventions targeting factors such as bullying, leadership practices or possibilities to learn new skills are arguably easier to implement, and it seems reasonable to assume that such interventions can also indirectly improve job satisfaction.

We have shown the results from two types of comparisons. The ‘dichotomous contrasts’ compared settings with the most versus least desirable covariate level. As such, these contrasts emulated the effects from a randomized controlled trial where a ‘treatment’ group is compared with a ‘non-treatment’ group. As these contrasts do not depend on the covariate levels in the data, the magnitudes of these contrasts are likely to be generalizable to other settings as an upper boundary of the possible effects of a given improvement. On the other hand, the ‘cohort-specific’ contrasts mimic a more realistic scenario by estimating the effects of improvements in comparison with actually observed covariate levels. However, the reference group to which the improvements are compared had covariate levels that are specific for this cohort. Therefore, these effects are likely only generalizable to settings with similar work environments such as other public healthcare enterprises in Denmark.

Importantly, however, neither of the estimated contrasts may be directly translated into expected outcomes of actual interventions. Real-world interventions require ample knowledge about interventional mechanisms, and for whom and under which circumstance they work, to achieve the desired outcomes (36). In this study, we considered rather simplistic scenarios in which improvements were simulated separately for each psychosocial work environment factor. Another approach would have been to collapse singular items into domains (for example leadership quality or overall job satisfaction). However, as evident from the results, different items within these domains had highly varying associations with turnover rates. Likewise, the scenarios considered states where all employees were set to experience the most desirable level of a given factor. Improvements of this magnitude would be difficult to achieve through actual interventions. However, by estimating these effects, our study suggests key areas for improvement, which may be used to inspire targets for future interventions. Finally, to closer mimic a multiple intervention scenario, we could have simulated smaller changes in several covariates simultaneously. This analysis was beyond the scope of this paper but would be interesting to pursue in future research.

### Strengths and limitations

Several strengths apply to our study. We estimated both dichotomous and cohort-specific contrasts, allowing researchers and policymakers to assess interventional potentials in both an experimental and a real-life setting. The large cohort with rich information on the psychosocial work environment and other factors allowed for adjustment for many confounders and estimation of occupational group-specific effects. Further, we used longitudinal data with clear temporal separation between baseline measures and subsequent turnover.

Some limitations of the study warrant discussion. First, we were not able to distinguish between voluntary and involuntary turnover (37). The majority of the turnover was likely voluntary, as the number of healthcare employees was stable throughout the follow-up period (38), indicating that no major layoffs occurred. Among the 2552 employees who left, 10% had been on long-term sickness absence during the follow-up period. Sickness absence and turnover have been found to share psychosocial work environment determinants (39), indicating that involuntary health-related turnover may also have been influenced by the psychosocial work environment, perhaps through the same mechanisms.

We measured turnover as having left the Capital Region of Denmark, and workplace shifts within the organization were not registered. Shifts between work units within the organization could only be measured indirectly through work-unit affiliations, and these affiliations could change for reasons other than turnover (for example, mergers or split-ups of units), which were not measured in the data. In contrast, turnover from the entire organization was directly measured and was therefore considered more reliable. A previous article using the same data found a one-year turnover rate from work units of 17% (9). Hence, we are likely to have underestimated the turnover rate compared with what was actually experienced in work units, and future research should address whether within-organization turnover share similar determinants.

Several factors that are associated with turnover, such as burnout (4, 12), adverse team climate (14), and organizational commitment (4) were not included in our data. Also, we did not have data on introductory programs for new employees, which have proven effective at reducing turnover in interventions (32). Furthermore, we did not have information on turnover-related factors external to the workplace, such as availability of alternative jobs, wage prospects in alternative jobs or family preferences (40).

Psychosocial work environment factors conceptually overlap and therefore tend to correlate (41). We did not correct for multicollinearity since we interpreted predicted outcomes (and not regression coefficients directly) and quantified uncertainty via bootstrapping. Survey responses of individual participants could also correlate, negatively or positively, because of, for example, psychological traits or mood states (42). We lacked data on, for example, personality traits to investigate the potential impact of this bias. Also, we did not account for multiple testing since we aimed to estimate effect sizes and to investigate whether patterns of effects were the same across subgroups rather than testing specific hypotheses.

We excluded 12 psychosocial working condition covariates to reduce overfitting by determining their association with the outcome. We discarded other approaches to covariate selection such as backwards elimination or more recent approaches such as the LASSO or elastic net algorithms since both of these approaches can be too strict, thereby eliminating important confounders (26). The exclusion cut-off was chosen to be explained model deviance = 0.25%, which we considered as conservative. Had we instead used, for example, a statistical significance (alpha = 0.05) criterion in the ANOVA analyses, we would have included only nine psychosocial work environment covariates.

Based on our conceptual framework, we assumed a unidirectional temporal direction from workplace characteristics through psychosocial working conditions and cognitive and emotional reactions. As all these covariates were measured simultaneously, we cannot rule out reciprocal associations between the covariates. Similarly, it is also possible that employees report poor work environments because of previous high rates of turnover in their unit (11).

### Concluding remarks

The one-year turnover rate was 10.5% in this cohort of hospital employees. We showed that improvements in the psychosocial work environment may hold great potential for reducing turnover rates among hospital staff, especially among nurses. The most prominent areas related to turnover included satisfaction with work prospects and general job satisfaction, as well as bullying, collegial respect for differences, organization of work and the possibility to learn new things.

### Funding

The Danish Regions (employer organization) and The Danish Association of Local Government Employees Organizations (Forhandlingsfællesskabet; employee organization) supported this work. Naja Hulvej Rod received funding from the Working Environment Foundation (grant no. 13-2015-09). The sponsors had no role in the study design, the collection, analysis and interpretation of the data, the writing of the report or the decision to submit it for publication.

### Conflicts of Interest

The authors declare no conflicts of interest.

### Protection of research participants

Research ethics approval is required from Danish authorities only when studying biological materials. As this study used only survey and administrative data, no research ethics approval was obtained.

## Supplementary material

Supplementary material
