# Supplementary material for: Reducing employee turnover in hospitals: estimating the effects of hypothetical improvements in the psychosocial work environment
Source: Scand J Work Environ Health. 2021 Aug 31;47(6):456–65. doi: 10.5271/sjweh.3969 (PMC8504546; doi:10.5271/sjweh.3969)
Supplement: Supplementary material [file SJWEH-47-456-S001.pdf]

Reducing employee turnover in hospitals: estimating the effects of hypothetical improvements in the psychosocial work environment[1]

by Jimmi Mathisen, MSc,[2] Tri-Long Nguyen, PhD, Johan Høy Jensen, PhD, Reiner Rugulies, PhD, Naja Hulvej Rod

1. *Supplementary Material*
2. *Correspondence to: Jimmi Mathisen, Department of Public Health, University of Copenhagen, Oester Farimagsgade 5, P.O. Box 2099, 1014 Copenhagen, Denmark. [E-mail: jima@sund.ku.dk] ORCID: 0000-0002-7391-7296*

Appendix 1. Details of the measurement and operationalization of psychosocial working conditions and cognitive and emotional reactions.

Supplementary Table S1. Domains, covariates, original questions, original scale, and COPSOQ-II status of all 39 covariates. Deviance explained refers to proportion of deviance explained in logistic regression using ANOVA type-II used for covariate selection (only for items in the psychosocial working conditions domain). Items marked in grey are included in the prediction models. Domains within psychosocial working conditions are denoted *(P)* and domains within cognitive and emotional reactions are denoted *(C)*.

Domain	No.	Covariate	Original question	Original scale	COPSOQ-II?	% deviance explained
			To what degree…			
Decision authority (P)	1	Influence on work	*… do you have influence on how you do your work?*	5 pt. Likert	X	0.416%
	2	Influence on schedule	*… are you able to schedule your work time, so that you can take into account private matters*	5 pt. Likert		0.297%
	3	Suggestions are taken serious	*… are your ideas and suggestions heard at your workplace?*	5 pt. Likert		0.277%
Job satisfaction (C)	4	General job satisfaction	*… are you pleased with your job as a whole, everything taken into consideration?*	7 pt. Likert	X	-
	5	Satisfaction with work prospects	*… are you pleased with your work prospects?*	7 pt. Likert	X	-
	6	Satisfaction with the use of abilities	*… are you pleased with the way your abilities are used?*	7 pt. Likert	X	-
	7	Satisfaction with work environment	*… are you pleased with the work environment?*	7 pt. Likert	X	-
Justice (P)	8	Conflicts are resolved in a fair way	*… are conflicts resolved in a fair way?*	7 pt. Likert	X	0.333%
	9	Work tasks are distributed fairly	*… is the work distributed fairly?*	7 pt. Likert	X	0.157%
Predictability (P)	10	Timely information on changes	*… are you informed well in advance concerning for example important decisions, changes, or plans for the future?*	5 pt. Likert	X	0.300%
Preventive efforts (P)	11	Adequate emotional strain support	*… does the workplace help the employees with managing emotionally disturbing situations at work?*	5 pt. Likert		0.443%
	12	Adequate stress prevention efforts	*… does the workplace focus enough on preventing stress in the employees?*	5 pt. Likert		0.251%
	13	Adequate stress support efforts	*… does the workplace help employees having problems with stress?*	5 pt. Likert		0.045%
Perceived quality of work (C)	14	Pride in work	*… are you proud of the work you and your colleagues do at your workplace?*	5 pt. Likert		-
	15	Satisfaction with quality of work	*… are you pleased with the quality of work you and your colleagues do at your workplace?*	5 pt. Likert		-
Recognition (P)	16	Recognition from the management	*… is your work recognized and appreciated by the management?*	5 pt. Likert	X	0.255%
Role clarity (P)	17	Clear objectives for work	*… does your work have clear objectives?*	5 pt. Likert	X	0.230%
Skill discretion (P)	18	Possibility to learn new things	*… do you have the possibility of learning new things through your work?*	5 pt. Likert	X	0.709%
Social capital (P)	19	Work unit social capital	Items 8, 9, 21, 22, 23, 24, 27, 28 (see operationalization note below)	-	-	0.310%
Social relations (P)	20	Collegial initiatives to improve work	*… are you and your colleagues good at coming up with suggestions for improving work procedures?*	5 pt. Likert		0.945%
	21	Collegial respect for differences	*… do you and your colleagues give space for each other's differences at your workplace? (e.g. regarding sex, age and background)*	5 pt. Likert		0.579%
	22	Takes responsibility for atmosphere	*… do you and your colleagues take responsibility for a nice atmosphere and tone of communication?*	5 pt. Likert		0.194%
	23	Staff groups respected by other staff groups	*… is your staff group respected by other staff groups at the workplace?*	5 pt. Likert		0.136%

Social support (P)	24	Social support from colleagues	*… do you get help and support from your colleagues when needed?*	5 pt. Likert	X	0.045%
	25	Social support from supervisor	*… do you get help and support by your nearest supervisor when needed?*	5 pt. Likert	X	0.249%
Perceived stress (C)	26	Stress within last 6 months	*… have you been stressed during the past six months?*	Yes (daily; weekly; monthly; sometimes) / No	X	-
Trust (P)	27	Trust in messages from management	*… can you trust the information that comes from the management?*	7 pt. Likert	X	0.318%
	28	Management trust employees	*… does the management trust the employees to do their work well?*	7 pt. Likert	X	0.124%
Work demands (C)	29	Able to have breaks during workday	*… do you have time for breaks throughout your workday?*	5 pt. Likert		0.488%
	30	Have time enough for tasks	*… do you have enough time for your work tasks?*	5 pt. Likert	X	0.253%
	31	Able to work without interruptions	*… are you able to work without being interrupted?*	5 pt. Likert		0.523%
			To what extent would you say that your immediate supervisor, <NAME>…			
Leadership (P)	32	Supervisor ability to organize work	*… is good at organizing work*	5 pt. Likert	X	0.596%
	33	Supervisor prioritizes workplace wellbeing	*… gives high priority to workplace wellbeing*	5 pt. Likert	X	0.289%
	34	Know who to ask about questions	*To what degree do you know whom to consult if you have questions regarding your work tasks?*	5 pt. Likert		0.043%
	35	Have had performance and development review within last 12 mo.	*Have you had a performance and development review during the past 12 months?*	Yes / No		1.815%
			Have you been exposed to…			
Offensive behaviors (P)	36	Bullying, last 12 mo.	*… bullying during the past 12 months?*	Yes (daily; weekly; monthly; sometimes) / No	X	1.326%
	37	Unwanted sexual attention, last 12 mo.	*… unwanted sexual attention at your workplace during the past 12 months?*	Yes (daily; weekly; monthly; sometimes) / No	X	0.246%
	38	Threats of violence, last 12 mo.	*… threats of violence at your workplace during the past 12 months?*	Yes (daily; weekly; monthly; sometimes) / No	X	0.070%
	39	Physical violence, last 12 mo.	*… physical violence at your workplace during the past 12 months?*	Yes (daily; weekly; monthly; sometimes) / No	X	0.134%

Operationalization:

- All items measured on 5-point Likert scales were categorized according to level of agreement: *Low (1-2); Medium (3); High (4-5)*.
- All items measured on 7-point Likert scales were categorized according to level of agreement: *Low (1-2); Medium (3-5); High (6-7)*.
- Items measuring offensive behaviors were dichotomized: No (No); Yes (Yes, daily + weekly + monthly + sometimes).
- Item 26 measuring perceived stress was categorized according to level of perceived stress: None (No); Low (Yes, sometimes + monthly); High (Yes, weekly + daily).
- Item 35 on whether a performance and development review had been held within the last 12 month was kept dichotomous: Yes; No
- Work-unit social capital (item 19) was calculated using eight items covering trust, justice and collaboration. These were re-computed into percentages, and the individual social capital scores was given by the mean of these percentages. The individual scores were then aggregated within work units, and assigned to all members of the work unit. The procedure has previously been used in studies of the WHALE cohort (9,23)

Appendix 2. Covariate distributions

Table S2. Distribution of psychosocial working conditions, and cognitive and emotional processes as well as exit rates within these strata. N=24 385

Psychosocial working conditions	Low		Medium		High		Do not know / not relevant	
	% N	% exit	% N	% exit	% N	% exit	% N	% exit
**Decision authority**								
Influence on work	6	17	31	12	62	9	0.3	-[a]
Influence on schedule	15	14	36	11	48	9	1	8
Suggestions are taken seriously	11	15	37	11	50	9	1	13
**Justice**								
Conflicts are resolved in a fair way	8	15	49	10	29	9	14	12
**Predictability**								
Timely information on changes	24	13	45	10	29	9	2	12
**Preventive efforts**								
Adequate emotional strain support	15	16	31	10	44	9	9	11
Adequate stress prevention efforts	39	13	39	9	13	8	10	10
**Recognition**								
Recognition from the management	9	16	24	12	63	9	5	15
**Skill discretion**								
Possibility to learn new things	9	15	31	10	61	10	0.2	18
**Social capital**								
Social capital in work unit	28	11	54	11	18	9	-	-
**Social relations**								
Collegial initiatives to improve work	4	14	34	12	60	10	1.4	14
Collegial respect for differences	4	17	22	12	73	10	1.1	12
**Trust**								
Trust in messages from management	6	15	49	11	42	9	3	12
**Work demands**								
Able to have breaks during workday	26	13	48	9	25	10	0.4	-[a]
Able to work without interruptions	45	12	40	9	15	11	0.5	-[a]
Have time enough for tasks	28	13	52	9	20	10	0.3	6
**Leadership**								
Supervisor ability to organize work	11	16	32	10	50	9	7	13
Supervisor prioritizes workplace wellbeing	10	16	26	11	58	9	6	13
	No		Yes				Do not know / not relevant	
Have had performance and development review within last 12 mo.	31	13	63	9	-	-	6	16
**Offensive behaviors**								
Bullying, last 12 mo.	88	10	10	15	-	-	1	16

Cognitive and emotional reactions	Low		Medium		High		Do not know / not relevant	
	% N	% exit	% N	% exit	% N	% exit	% N	% exit
**Job satisfaction**								
General job satisfaction	3	24	39	13	57	8	0.6	11
Satisfaction with work prospects	10	20	45	11	41	7	4	16
Satisfaction with the use of abilities	7	20	45	12	47	8	0.9	14
Satisfaction with work environment	12	17	53	11	35	8	0.7	11
**Perceived quality of work**								
Pride in work	5	18	28	14	67	9	0.6	7
Satisfaction with quality of work	7	18	33	12	59	9	0.5	13
	None		Low		High		Do not know / not relevant	
**Perceived stress**								
Stress within last six months	27	8	47	10	25	14	2	13

a Not reported due to small n

Appendix 3. Performance of prediction models

The performance of the overall prediction models used for estimation are summarized in Figure S1 panel A and B. The model predicting turnover from psychosocial working conditions adjusted for sociodemographic factors and workplace and employment characteristics had a concordance statistic of 0.74 (95% CI: 0.73–0.75) (Panel A). The model predicting turnover from cognitive and emotional reactions, sociodemographic factors and workplace and employment characteristics and psychosocial working conditions had a concordance statistic of 0.76 (95% CI: 0.75–0.77) (Panel B). These concordance statistics indicate good discriminative performance, that is, the models were good at discriminating between employees who do and who do not leave the Capital Region of Denmark. The slopes of 1.26 indicate that the models are conservative in the sense that they predict fewer outcomes than expected. As can be seen from the plots, this conservativism increases as the predicted probability increases. However, as can also be seen from the plots, the majority of the employees had predicted probabilities of turnover between 0 and 0.20. In this range, the predicted probabilities were close to the observed probabilities.

Figure S1. Predictive performance of the prediction models used for estimation. Panel A shows the performance from the model predicting turnover from psychosocial working conditions adjusted for sociodemographic factors and workplace and employment characteristics. Panel B shows the performance of the model predicting turnover from cognitive and emotional reactions, sociodemographic factors and workplace and employment characteristics and psychosocial working conditions.

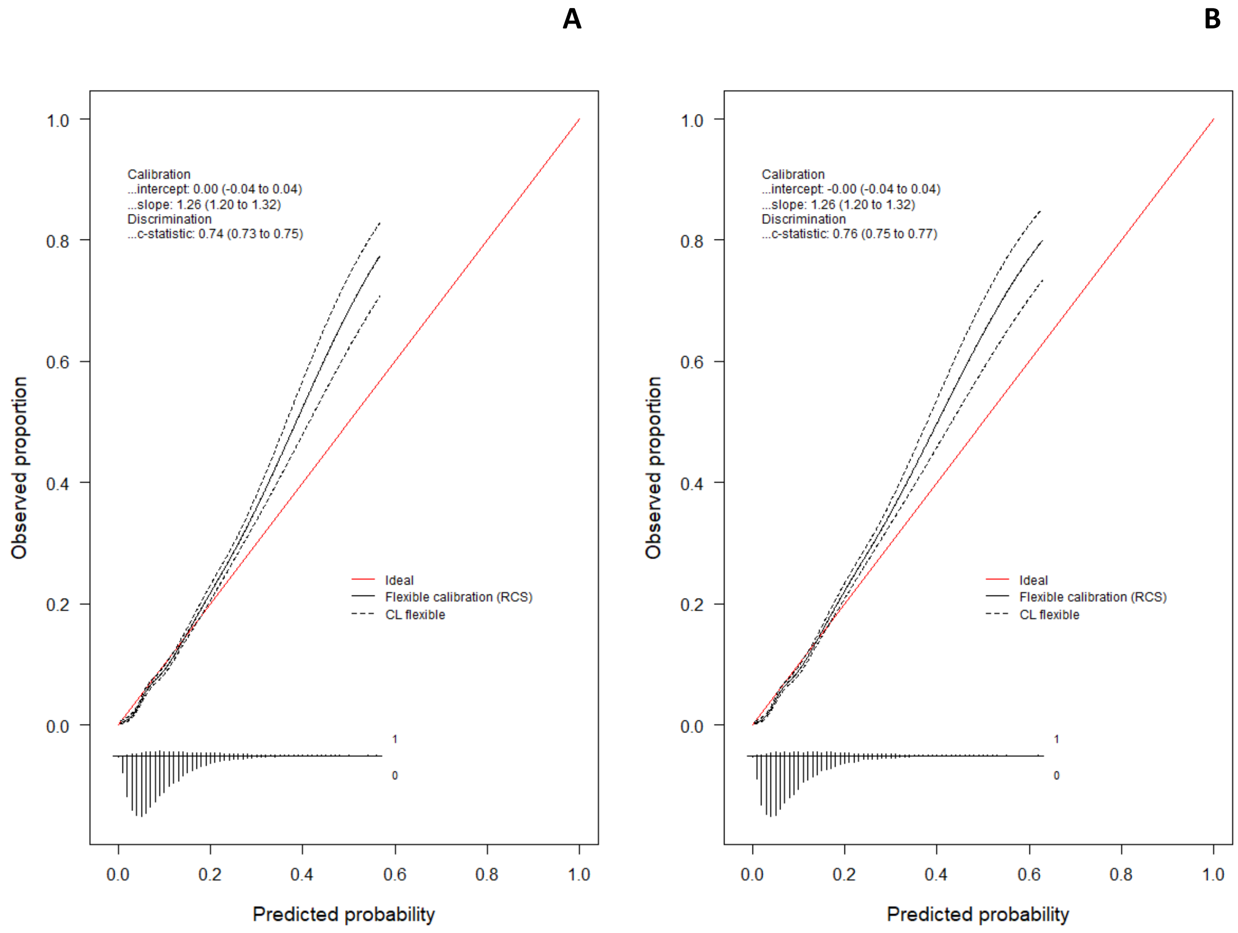

Appendix 4. Internal-external cross-validation of prediction models

The summary performance of the internal-external cross-validation models is shown in the figures below. Figure S2 shows the summary concordance statistics of models predicting turnover across the 13 organizations from psychosocial working conditions along with sociodemographic factors and workplace and employment characteristics. These models had a summary c-statistic of 0.68 (95% CI: 0.65–0.71). Figure S3 shows summary concordance statistics of models predicting turnover across the 13 organizations from cognitive and emotional reactions along with sociodemographic factors, workplace and employment characteristics and psychosocial working conditions. These models had a summary c-statistic of 0.70 (95% CI: 0.67; 0.73). The summary c-statistics indicate that the models have fairly good ability to discriminate between those who do and those who do not leave across the 13 organizations.

The plots indicate some degree of heterogeneity in discriminative ability. However, as indicated by the confidence bars, some organizations had few employees and few events (e.g. organization 4 (n = 432, events = 26), organization 5 (n=173, events = 43) and organization 12 (n = 385, events = 50).

Figure S2. Meta-analysis of concordance statistic and 95% confidence intervals of internal-external validation models across 13 organizations. The models predicted turnover from psychosocial working conditions adjusted for sociodemographic factors and workplace and employment characteristics. The dotted line indicates the concordance statistic of the overall model with the same covariates (Figure S1 Panel A).

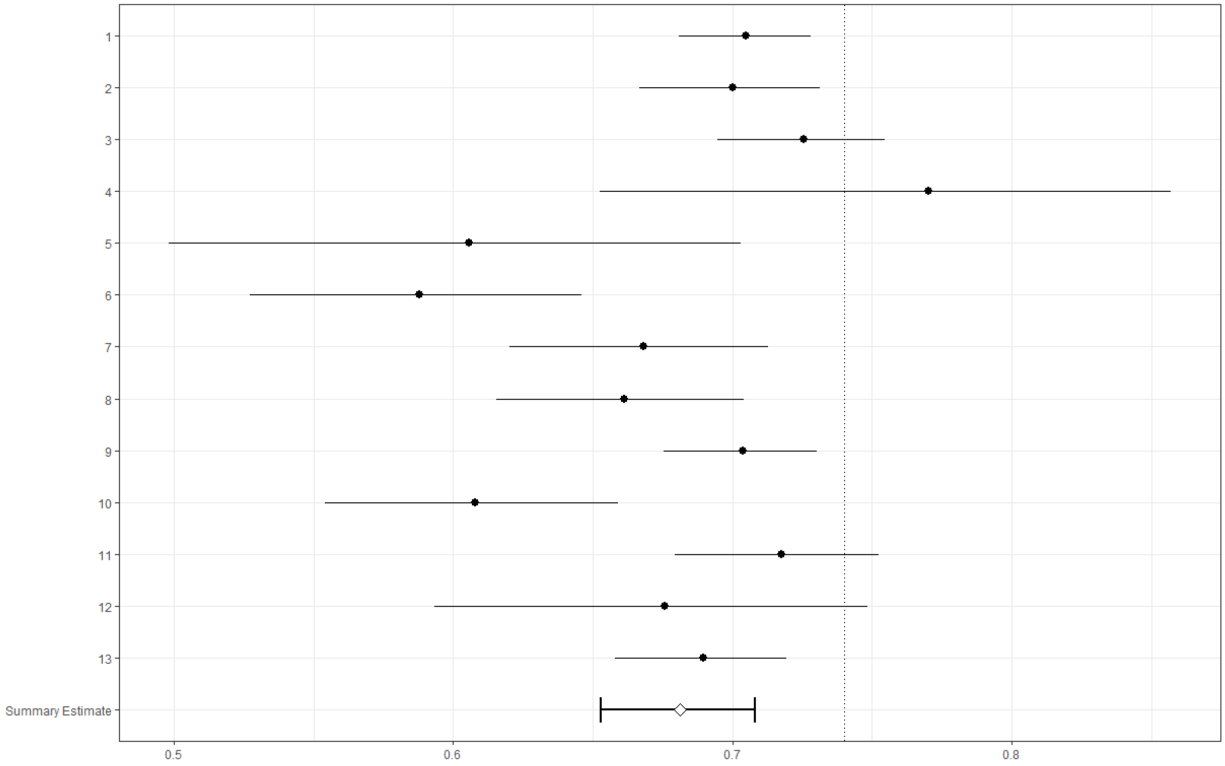

Figure S3. Meta-analysis of concordance statistic and 95% confidence intervals of internal-external validation models across 13 organizations. The models predicted turnover from cognitive and emotional reactions, sociodemographic factors and workplace and employment characteristics and psychosocial working conditions. The dotted line indicates the concordance statistic of the overall model with the same covariates (Figure S1 Panel B).

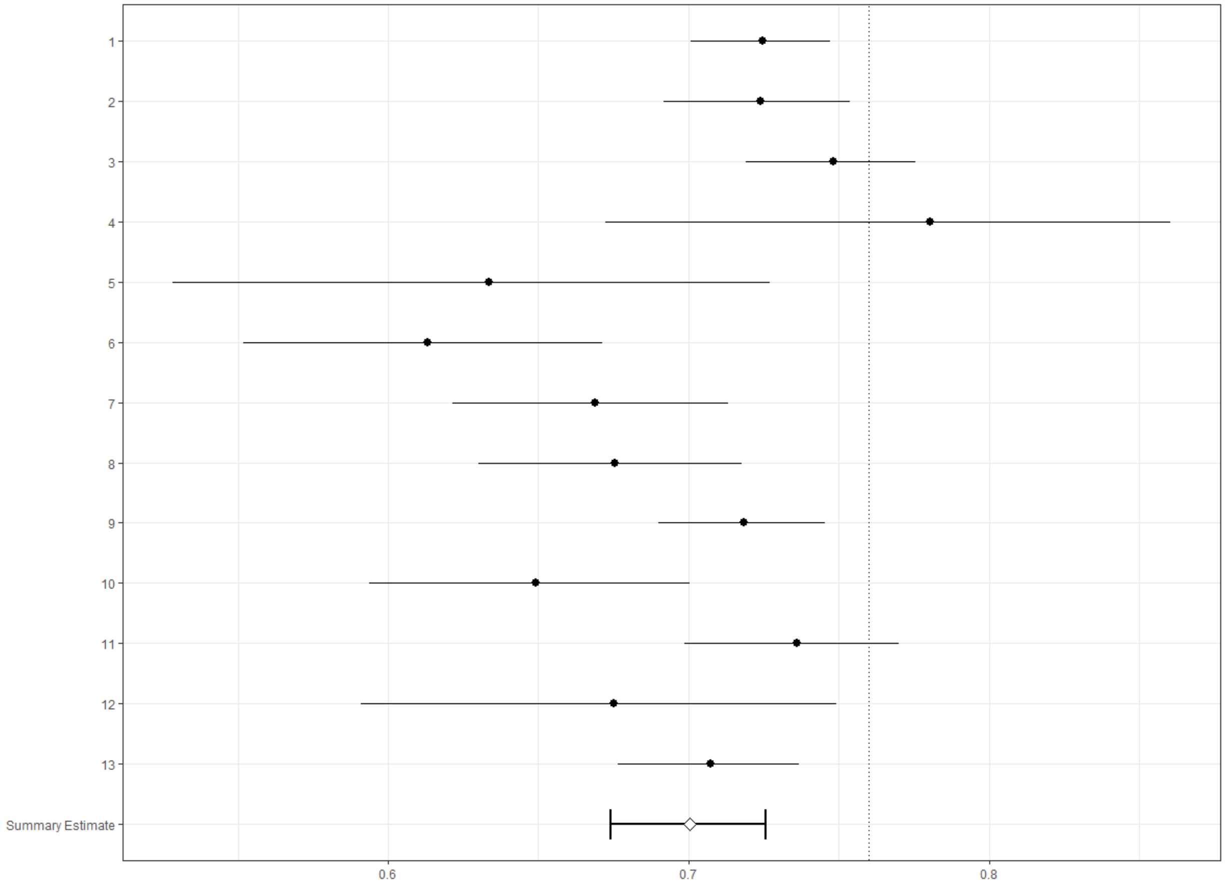

Appendix 5: Estimates associations for all employees combined as well as for each occupational group (Table S3 – S10)

Table S3. All employees (n=24 385). Rate differences in turnover per 10 000 employees per year and 95% confidence intervals. Dichotomous and cohort-specific contrasts resulting from hypothetical improvements in psychosocial working conditions and cognitive and emotional reactions.

	Estimated rate of turnover	95% CI Low	95% CI High
**No intervention**	1047	1037	1056

	Dichotomous contrasts RD	95% CI Low	95% CI High	Cohort-specific contrasts RD	95% CI Low	95% CI High
**All interventions simultaneously**	-2135	-2151	-2120	-456	-467	-445

**Individual interventions**		Dichotomous contrasts RD	95% CI Low	95% CI High	Cohort-specific contrasts RD	95% CI Low	95% CI High
**Psychosocial working conditions[a]**							
**Domain**	**Item**						
Decision authority	Influence on work	-76	-89	-62	-19	-33	-6
	Influence on schedule	-50	-63	-37	-18	-31	-5
	Suggestions are taken seriously	-86	-100	-73	-28	-41	-14
Justice	Conflicts are resolved in a fair way	-64	-78	-51	-21	-34	-8
Leadership	Have had performance and development review within last 12 months	-92	-106	-79	-39	-52	-26
	Supervisor ability to organize work	-138	-151	-124	-25	-38	-12
	Supervisor prioritizes workplace wellbeing	-71	-85	-57	-15	-28	-2
Offensive behaviors	Bullied within last 12 months	-200	-214	-186	-26	-39	-12
Predictability	Timely information on changes	-35	-49	-22	1	-13	14
Preventive efforts	Adequate emotional strain support	-109	-123	-96	-9	-22	5
	Adequate stress prevention efforts	-41	-55	-28	-29	-42	-16
Recognition	Recognition from the management	-113	-126	-99	-35	-48	-22
Skill discretion	Possibility to learn new things	-129	-142	-115	-16	-30	-3
Social capital	Social capital in work unit	-12	-26	1	-26	-39	-12
Social relations	Collegial initiatives to improve work	-71	-84	-58	-44	-57	-31
	Collegial respect for differences	-131	-144	-118	-33	-46	-20
Trust	Trust in messages from management	-102	-116	-89	-33	-46	-20
Work demands	Able to have breaks during workday	-51	-65	-37	11	-2	24
	Able to work without interruptions	17	3	30	35	22	49
	Have time enough for tasks	-58	-72	-45	-19	-32	-5
**Cognitive and emotional reactions[b]**							
**Domain**	**Item**						
Job satisfaction	Satisfaction with work prospects	-522	-536	-508	-145	-159	-132
	Satisfaction with the use of abilities	-71	-86	-57	24	10	38
	Satisfaction with the work environment	-43	-58	-29	0	-14	15
	General job satisfaction	-339	-353	-325	-121	-134	-108
Perceived stress	Stress within last 6 months	-118	-132	-104	-49	-62	-35
Perceived quality of work	Pride in work	-56	-70	-42	-39	-53	-25
	Satisfaction with quality of work	-43	-58	-29	-5	-20	9

a Adjusted for sociodemographic structures and workplace and employment characteristics.

b Adjusted for sociodemographic structures, workplace and employment characteristics, and psychosocial working conditions

Table S4. Physicians (n=2154). Rate differences in turnover per 10 000 employees per year and 95% confidence intervals. Dichotomous and cohort-specific contrasts resulting from hypothetical improvements in psychosocial working conditions and cognitive and emotional reactions.

	Estimated rate of turnover	95% CI Low	95% CI High
**No intervention**	1365	1327	1403

	Dichotomous contrasts RD	95% CI Low	95% CI High	Cohort-specific contrasts RD	95% CI Low	95% CI High
**All interventions simultaneously**	-2118	-2177	-2060	-441	-484	-397

**Individual interventions**		Dichotomous contrasts RD	95% CI Low	95% CI High	Cohort-specific contrasts RD	95% CI Low	95% CI High
**Psychosocial working conditions[a]**							
**Domain**	**Item**						
Decision authority	Influence on work	-103	-155	-50	-36	-88	16
	Influence on schedule	-60	-112	-8	-31	-82	20
	Suggestions are taken seriously	-95	-148	-43	-29	-80	23
Justice	Conflicts are resolved in a fair way	-76	-129	-24	-24	-76	28
Leadership	Have had performance and development review within last 12 months	-126	-177	-74	-63	-114	-11
	Supervisor ability to organize work	-84	-138	-30	11	-42	64
	Supervisor prioritizes workplace wellbeing	-95	-148	-42	-21	-74	31
Offensive behaviors	Bullied within last 12 months	-302	-357	-246	-31	-83	21
Predictability	Timely information on changes	-33	-86	20	9	-44	61
Preventive efforts	Adequate emotional strain support	-158	-212	-104	-14	-67	38
	Adequate stress prevention efforts	-28	-80	24	-19	-71	33
Recognition	Recognition from the management	-194	-247	-142	-57	-108	-5
Skill discretion	Possibility to learn new things	-140	-194	-86	-3	-56	49
Social capital	Social capital in work unit	19	-32	70	-37	-89	15
Social relations	Collegial initiatives to improve work	-122	-172	-72	-96	-146	-45
	Collegial respect for differences	-178	-230	-125	-47	-99	5
Trust	Trust in messages from management	-131	-183	-79	-26	-78	26
Work demands	Able to have breaks during workday	30	-23	83	23	-31	76
	Able to work without interruptions	10	-42	63	21	-32	73
	Have time enough for tasks	19	-33	71	-3	-55	50
**Cognitive and emotional reactions[b]**							
**Domain**	**Item**						
Job satisfaction	Satisfaction with work prospects	-528	-584	-472	-57	-108	-6
	Satisfaction with the use of abilities	-103	-157	-49	25	-27	78
	Satisfaction with the work environment	-55	-108	-2	3	-49	56
	General job satisfaction	-260	-313	-207	-58	-109	-7
Perceived stress	Stress within last 6 months	-69	-121	-16	-26	-77	26
Perceived quality of work	Pride in work	-72	-123	-20	-38	-90	14
	Satisfaction with quality of work	3	-51	56	22	-31	75

a Adjusted for sociodemographic structures and workplace and employment characteristics.

b Adjusted for sociodemographic structures, workplace and employment characteristics, and psychosocial working conditions

Table S5. Nurses (n=8768). Rate differences in turnover per 10 000 employees per year and 95% confidence intervals. Dichotomous and Cohort-specific contrasts resulting from hypothetical improvements in psychosocial working conditions and cognitive and emotional reactions.

	Estimated rate of turnover	95% CI Low	95% CI High
**No intervention**	1073	1055	1091

	Dichotomous contrasts RD	95% CI Low	95% CI High	Cohort-specific contrasts RD	95% CI Low	95% CI High
**All interventions simultaneously**	-2357	-2383	-2331	-521	-539	-503

**Individual interventions**		Dichotomous contrasts RD	95% CI Low	95% CI High	Cohort-specific contrasts RD	95% CI Low	95% CI High
**Psychosocial working conditions[a]**							
**Domain**	**Item**						
Decision authority	Influence on work	-79	-101	-57	-22	-44	-1
	Influence on schedule	-52	-74	-30	-18	-40	3
	Suggestions are taken seriously	-87	-108	-65	-27	-48	-6
Justice	Conflicts are resolved in a fair way	-65	-86	-44	-21	-42	0
Leadership	Have had performance and development review within last 12 months	-98	-119	-76	-44	-65	-23
	Supervisor ability to organize work	-175	-197	-153	-21	-42	1
	Supervisor prioritizes workplace wellbeing	-72	-94	-50	-12	-34	9
Offensive behaviors	Bullied within last 12 months	-184	-207	-162	-24	-45	-2
Predictability	Timely information on changes	-48	-70	-26	-9	-30	13
Preventive efforts	Adequate emotional strain support	-114	-136	-92	-16	-37	6
	Adequate stress prevention efforts	-73	-94	-52	-59	-81	-38
Recognition	Recognition from the management	-99	-121	-77	-31	-53	-10
Skill discretion	Possibility to learn new things	-131	-153	-108	-12	-34	10
Social capital	Social capital in work unit	-21	-43	0	-24	-46	-3
Social relations	Collegial initiatives to improve work	-70	-92	-49	-38	-59	-17
	Collegial respect for differences	-129	-151	-108	-32	-54	-11
Trust	Trust in messages from management	-97	-118	-75	-33	-55	-12
Work demands	Able to have breaks during workday	-89	-111	-67	-30	-51	-8
	Able to work without interruptions	21	-1	44	46	24	68
	Have time enough for tasks	-101	-122	-79	-33	-55	-12
**Cognitive and emotional reactions[b]**							
**Domain**	**Item**						
Job satisfaction	Satisfaction with work prospects	-539	-563	-515	-151	-173	-129
	Satisfaction with the use of abilities	-69	-94	-45	27	2	51
	Satisfaction with the work environment	-44	-68	-20	-1	-25	22
	General job satisfaction	-390	-413	-367	-152	-174	-130
Perceived stress	Stress within last 6 months	-150	-173	-126	-62	-85	-39
Perceived quality of work	Pride in work	-56	-79	-32	-42	-66	-19
	Satisfaction with quality of work	-91	-115	-68	-29	-52	-5

a Adjusted for sociodemographic structures and workplace and employment characteristics.

b Adjusted for sociodemographic structures, workplace and employment characteristics, and psychosocial working conditions

Table S6. Other healthcare employees (n=5507). Rate differences in turnover per 10 000 employees per year and 95% confidence intervals. Dichotomous and cohort-specific contrasts resulting from hypothetical improvements in psychosocial working conditions and cognitive and emotional reactions.

	Estimated rate of turnover	95% CI Low	95% CI High
**No intervention**	946	927	964

	Dichotomous contrasts RD	95% CI Low	95% CI High	Cohort-specific contrasts RD	95% CI Low	95% CI High
**All interventions simultaneously**	-1742	-1772	-1711	-388	-408	-368

**Individual interventions**		**Dichotomous contrasts**			**Cohort-specific contrasts**		
		**RD**	**95% CI Low**	**95% CI High**	**RD**	**95% CI Low**	**95% CI High**
**Psychosocial working conditions**[a]							
**Domain**	**Item**						
Decision authority	Influence on work	-68	-93	-43	-18	-42	7
	Influence on schedule	-46	-70	-22	-18	-42	6
	Suggestions are taken seriously	-82	-106	-58	-29	-52	-5
Justice	Conflicts are resolved in a fair way	-59	-83	-35	-19	-43	5
Leadership	Have had performance and development review within last 12 months	-80	-104	-56	-34	-58	-10
	Supervisor ability to organize work	-124	-148	-99	-35	-58	-11
	Supervisor prioritizes workplace wellbeing	-63	-87	-38	-14	-38	10
Offensive behaviors	Bullied within last 12 months	-177	-202	-151	-25	-49	-1
Predictability	Timely information on changes	-28	-53	-3	0	-24	25
Preventive efforts	Adequate emotional strain support	-87	-112	-61	-3	-28	21
	Adequate stress prevention efforts	-10	-34	15	-12	-37	12
Recognition	Recognition from the management	-98	-122	-73	-32	-56	-8
Skill discretion	Possibility to learn new things	-121	-146	-96	-18	-42	7
Social capital	Social capital in work unit	-13	-37	12	-19	-43	6
Social relations	Collegial initiatives to improve work	-60	-84	-36	-34	-58	-10
	Collegial respect for differences	-118	-142	-93	-31	-55	-6
Trust	Trust in messages from management	-94	-119	-70	-34	-58	-10
Work demands	Able to have breaks during workday	12	-13	38	58	32	83
	Able to work without interruptions	16	-9	41	37	12	62
	Have time enough for tasks	-20	-45	5	-3	-28	21
**Cognitive and emotional reactions**[b]							
**Domain**	**Item**						
Job satisfaction	Satisfaction with work prospects	-476	-501	-450	-149	-173	-126
	Satisfaction with the use of abilities	-64	-91	-38	21	-5	47
	Satisfaction with the work environment	-39	-65	-14	0	-26	25
	General job satisfaction	-295	-320	-270	-105	-129	-81
Perceived stress	Stress within last 6 months	-71	-96	-45	-29	-54	-4
Perceived quality of work	Pride in work	-50	-75	-25	-35	-60	-10
	Satisfaction with quality of work	-5	-30	21	11	-14	37

a Adjusted for sociodemographic structures and workplace and employment characteristics.

b Adjusted for sociodemographic structures, workplace and employment characteristics, and psychosocial working conditions

Table S7. Pedagogical employees (n=587). Rate differences in turnover per 10 000 employees per year and 95% confidence intervals. Dichotomous and cohort-specific contrasts resulting from hypothetical improvements in psychosocial working conditions and cognitive and emotional reactions.

**No intervention**	**Estimated rate of turnover**	**95% CI Low**	**95% CI High**
	1278	1220	1337

**All interventions simultaneously**	**Dichotomous contrasts RD**	**95% CI Low**	**95% CI High**	**Cohort-specific contrasts RD**	**95% CI Low**	**95% CI High**
	-2064	-2151	-1978	-373	-438	-308

**Individual interventions**		**Dichotomous contrasts RD**	**95% CI Low**	**95% CI High**	**Cohort-specific contrasts RD**	**95% CI Low**	**95% CI High**
**Psychosocial working conditions[a]**							
**Domain**	**Item**						
Decision authority	Influence on work	-92	-164	-20	-20	-91	52
	Influence on schedule	-59	-130	11	-25	-95	45
	Suggestions are taken seriously	-107	-178	-35	-39	-109	30
Justice	Conflicts are resolved in a fair way	-76	-148	-5	-31	-102	41
Leadership	Have had performance and development review within last 12 months	-16	-87	56	-7	-79	66
	Supervisor ability to organize work	-138	-211	-65	-30	-102	41
	Supervisor prioritizes workplace wellbeing	-54	-128	19	-5	-77	68
Offensive behaviors	Bullied within last 12 months	-230	-305	-155	-43	-114	28
Predictability	Timely information on changes	-36	-108	37	5	-68	77
Preventive efforts	Adequate emotional strain support	-68	-142	7	31	-42	104
	Adequate stress prevention efforts	-13	-85	59	-5	-77	67
Recognition	Recognition from the management	-130	-203	-57	-27	-99	45
Skill discretion	Possibility to learn new things	-160	-233	-87	-19	-91	53
Social capital	Social capital in work unit	-17	-89	55	-22	-94	50
Social relations	Collegial initiatives to improve work	-85	-155	-16	-51	-121	19
	Collegial respect for differences	-172	-243	-102	-42	-113	29
Trust	Trust in messages from management	-137	-209	-65	-43	-115	28
Work demands	Able to have breaks during workday	36	-39	111	56	-17	130
	Able to work without interruptions	19	-53	92	41	-32	114
	Have time enough for tasks	-27	-99	46	16	-56	88
**Cognitive and emotional reactions[b]**							
**Domain**	**Item**						
Job satisfaction	Satisfaction with work prospects	-598	-676	-520	-124	-199	-49
	Satisfaction with the use of abilities	-58	-141	25	49	-33	131
	Satisfaction with the work environment	-47	-128	34	5	-75	86
	General job satisfaction	-391	-469	-312	-130	-205	-55
Perceived stress	Stress within last 6 months	-100	-180	-19	-26	-105	52
Perceived quality of work	Pride in work	-68	-145	10	-42	-121	36
	Satisfaction with quality of work	-39	-120	42	9	-71	90

a Adjusted for sociodemographic structures and workplace and employment characteristics.

b Adjusted for sociodemographic structures, workplace and employment characteristics, and psychosocial working conditions

Table S8. Service personnel (n=2647). Rate differences in turnover per 10 000 employees per year and 95% confidence intervals. Dichotomous and cohort-specific contrasts resulting from hypothetical improvements in psychosocial working conditions and cognitive and emotional reactions.

	Estimated rate of turnover	95% CI Low	95% CI High
**No intervention**	840	813	867

	Dichotomous contrasts RD	95% CI Low	95% CI High	Cohort-specific contrasts RD	95% CI Low	95% CI High
**All interventions simultaneously**	-1829	-1878	-1781	-322	-352	-291

**Individual interventions**		Dichotomous contrasts RD	95% CI Low	95% CI High	Cohort-specific contrasts RD	95% CI Low	95% CI High
**Psychosocial working conditions[a]**							
**Domain**	**Item**						
Decision authority	Influence on work	-55	-92	-19	-11	-47	25
	Influence on schedule	-40	-76	-4	-16	-51	19
	Suggestions are taken seriously	-73	-108	-37	-30	-65	5
Justice	Conflicts are resolved in a fair way	-54	-91	-18	-14	-50	22
Leadership	Have had performance and development review within last 12 months	-84	-120	-48	-29	-65	6
	Supervisor ability to organize work	-97	-134	-60	-14	-50	21
	Supervisor prioritizes workplace wellbeing	-56	-93	-20	-15	-51	21
Offensive behaviors	Bullied within last 12 months	-194	-231	-156	-33	-69	2
Predictability	Timely information on changes	-36	-72	0	-2	-38	34
Preventive efforts	Adequate emotional strain support	-96	-133	-59	-7	-43	28
	Adequate stress prevention efforts	-24	-60	12	-7	-42	29
Recognition	Recognition from the management	-111	-147	-75	-38	-73	-3
Skill discretion	Possibility to learn new things	-105	-142	-68	-25	-60	11
Social capital	Social capital in work unit	-3	-38	33	-17	-53	19
Social relations	Collegial initiatives to improve work	-66	-101	-31	-48	-83	-13
	Collegial respect for differences	-105	-141	-68	-31	-66	5
Trust	Trust in messages from management	-90	-125	-54	-30	-66	5
Work demands	Able to have breaks during workday	-44	-83	-6	65	28	102
	Able to work without interruptions	10	-27	46	17	-19	54
	Have time enough for tasks	-37	-73	0	-8	-44	28
**Cognitive and emotional reactions[b]**							
**Domain**	**Item**						
Job satisfaction	Satisfaction with work prospects	-415	-454	-377	-109	-144	-75
	Satisfaction with the use of abilities	-58	-97	-19	14	-24	53
	Satisfaction with the work environment	-35	-73	3	-1	-38	37
	General job satisfaction	-273	-311	-234	-79	-115	-44
Perceived stress	Stress within last 6 months	-66	-104	-29	-25	-62	12
Perceived quality of work	Pride in work	-46	-83	-8	-26	-64	11
	Satisfaction with quality of work	11	-27	50	21	-18	59

a Adjusted for sociodemographic structures and workplace and employment characteristics.

b Adjusted for sociodemographic structures, workplace and employment characteristics, and psychosocial working conditions

Table S9. Administrative leaders (n=292). Rate differences in turnover per 10 000 employees per year and 95% confidence intervals. Dichotomous and cohort-specific contrasts resulting from hypothetical improvements in psychosocial working conditions and cognitive and emotional reactions.

	Estimated rate of turnover	95% CI	
**No intervention**		**Low**	**High**
	800	742	858

	Dichotomous contrasts			Cohort-specific contrasts		
	**RD**	**95% CI**		**RD**	**95% CI**	
**All interventions simultaneously**		**Low**	**High**		**Low**	**High**
	-1859	-1964	-1753	-207	-270	-145

**Individual interventions**		**Dichotomous contrasts**			**Cohort-specific contrasts**		
		**RD**	**95% CI**		**RD**	**95% CI**	
			**Low**	**High**		**Low**	**High**
**Psychosocial working conditions[a]**							
**Domain**	**Item**						
Decision authority	Influence on work	-70	-147	7	-3	-79	72
	Influence on schedule	-32	-109	45	-4	-78	71
	Suggestions are taken seriously	-64	-142	14	-8	-83	67
Justice	Conflicts are resolved in a fair way	-57	-129	16	-25	-98	48
Leadership	Have had performance and development review within last 12 months	-92	-168	-16	-24	-98	51
	Supervisor ability to organize work	-97	-173	-20	-20	-94	54
	Supervisor prioritizes workplace wellbeing	-66	-143	10	-13	-88	62
Offensive behaviors	Bullied within last 12 months	-191	-270	-112	-14	-88	60
Predictability	Timely information on changes	-22	-99	55	11	-65	87
Preventive efforts	Adequate emotional strain support	-108	-186	-30	0	-75	75
	Adequate stress prevention efforts	-9	-85	67	-2	-77	73
Recognition	Recognition from the management	-121	-197	-45	-21	-95	53
Skill discretion	Possibility to learn new things	-133	-212	-54	-11	-86	65
Social capital	Social capital in work unit	-19	-91	52	-41	-114	32
Social relations	Collegial initiatives to improve work	-53	-127	22	-23	-97	51
	Collegial respect for differences	-145	-223	-67	-14	-89	61
Trust	Trust in messages from management	-111	-188	-34	-15	-89	59
Work demands	Able to have breaks during workday	-13	-91	65	22	-55	99
	Able to work without interruptions	14	-62	90	22	-54	98
	Have time enough for tasks	-17	-92	58	-3	-78	72
**Cognitive and emotional reactions[b]**							
**Domain**	**Item**						
Job satisfaction	Satisfaction with work prospects	-483	-563	-402	-89	-161	-16
	Satisfaction with the use of abilities	-74	-158	10	19	-62	101
	Satisfaction with the work environment	-39	-120	42	4	-76	83
	General job satisfaction	-243	-319	-166	-69	-142	5
Perceived stress	Stress within last 6 months	-61	-140	18	-11	-88	66
Perceived quality of work	Pride in work	-53	-130	24	-30	-107	47
	Satisfaction with quality of work	-9	-90	71	8	-72	88

a Adjusted for sociodemographic structures and workplace and employment characteristics.

b Adjusted for sociodemographic structures, workplace and employment characteristics, and psychosocial working conditions

Table S10. Administrative employees (n=4430). Rate differences in turnover per 10 000 employees per year and 95% confidence intervals. Dichotomous and cohort-specific contrasts resulting from hypothetical improvements in psychosocial working conditions and cognitive and emotional reactions.

	Estimated rate of turnover	95% CI Low	95% CI High
**No intervention**	1074	1053	1095

	Dichotomous contrasts RD	95% CI Low	95% CI High	Cohort-specific contrasts RD	95% CI Low	95% CI High
**All interventions simultaneously**	-2405	-2439	-2370	-526	-549	-503

**Individual interventions**		Dichotomous contrasts RD	95% CI Low	95% CI High	Cohort-specific contrasts RD	95% CI Low	95% CI High
**Psychosocial working conditions[a]**							
**Domain**	**Item**						
Decision authority	Influence on work	-76	-103	-48	-13	-40	15
	Influence on schedule	-52	-80	-24	-12	-39	15
	Suggestions are taken seriously	-94	-121	-66	-25	-52	1
Justice	Conflicts are resolved in a fair way	-69	-96	-41	-25	-52	2
Leadership	Have had performance and development review within last 12 months	-96	-123	-69	-36	-63	-8
	Supervisor ability to organize work	-134	-162	-107	-44	-71	-17
	Supervisor prioritizes workplace wellbeing	-79	-106	-51	-21	-48	7
Offensive behaviors	Bullied within last 12 months	-210	-239	-182	-22	-49	5
Predictability	Timely information on changes	-20	-48	7	15	-13	43
Preventive efforts	Adequate emotional strain support	-119	-147	-91	-4	-32	23
	Adequate stress prevention efforts	-41	-68	-13	-13	-40	14
Recognition	Recognition from the management	-116	-144	-89	-35	-62	-8
Skill discretion	Possibility to learn new things	-138	-166	-110	-24	-52	3
Social capital	Social capital in work unit	-15	-42	12	-36	-64	-9
Social relations	Collegial initiatives to improve work	-63	-90	-36	-40	-67	-13
	Collegial respect for differences	-137	-164	-110	-33	-60	-6
Trust	Trust in messages from management	-113	-140	-86	-36	-63	-9
Work demands	Able to have breaks during workday	-113	-141	-84	-11	-39	16
	Able to work without interruptions	16	-12	43	32	4	59
	Have time enough for tasks	-79	-106	-52	-29	-56	-2
**Cognitive and emotional reactions[b]**							
**Domain**	**Item**						
Job satisfaction	Satisfaction with work prospects	-601	-630	-572	-200	-228	-173
	Satisfaction with the use of abilities	-77	-110	-45	24	-7	55
	Satisfaction with the work environment	-46	-77	-14	3	-28	34
	General job satisfaction	-368	-398	-339	-138	-166	-109
Perceived stress	Stress within last 6 months	-174	-204	-143	-77	-107	-48
Perceived quality of work	Pride in work	-60	-90	-29	-45	-75	-14
	Satisfaction with quality of work	-55	-86	-24	-12	-43	19

a Adjusted for sociodemographic structures and workplace characteristics and employment.

b Adjusted for sociodemographic structures, workplace and employment characteristics, and psychosocial working conditions
